# Colour categories are reflected in sensory stages of colour perception when stimulus issues are resolved

**DOI:** 10.1371/journal.pone.0178097

**Published:** 2017-05-25

**Authors:** Lewis Forder, Xun He, Anna Franklin

**Affiliations:** 1 The Sussex Colour Group, School of Psychology, University of Sussex, Falmer, United Kingdom; 2 Cognition and Cognitive Neuroscience Research Centre, Department of Psychology, Bournemouth University, Poole, United Kingdom; Universidad de Salamanca, SPAIN

## Abstract

Debate exists about the time course of the effect of colour categories on visual processing. We investigated the effect of colour categories for two groups who differed in whether they categorised a blue-green boundary colour as the same- or different-category to a reliably-named blue colour and a reliably-named green colour. Colour differences were equated in just-noticeable differences to be equally discriminable. We analysed event-related potentials for these colours elicited on a passive visual oddball task and investigated the time course of categorical effects on colour processing. Support for category effects was found 100 ms after stimulus onset, and over frontal sites around 250 ms, suggesting that colour naming affects both early sensory and later stages of chromatic processing.

## Introduction

We perceive millions of different colours [1], but we necessarily use a limited number of colour terms (categories) to describe them (‘blue’, ‘yellow’, ‘orange’, etc.). There has been much debate on whether our naming of colour affects how we see it [2,3]. A common assertion is that colours are more easily distinguished if they are named with different terms (different-category) than with the same term (same-category) even if the colour differences are equated in some kind of colour metric [4–6]. There are many studies that report evidence for these so called ‘colour category effects’ on behavioural tasks where observers judge the difference between colours, memorize colours, or search for colours [7–9]. There has been recent discussion on whether these effects are reliable as some studies have failed to replicate colour category effects [10,11]. However, one crucial question is the extent to which colour terms affect how we perceive colour and the time course of any effect. Colour terms may simply affect ‘post-perceptual’ processes such as attention, task strategy or the stage of decision making [12,13]. Alternatively, colour terms may affect early sensory stages of colour processing [14,15]. The distinction between early and late effects of language on perception relates directly to debate about the way that perception may be penetrable by cognitive systems, such as language [16–18].

The argument that language affects sensory stages of processing has found support in domains other than colour [19,20]. However, for colour, the evidence is currently mixed. One approach to investigating this issue has been to employ the event-related potential (ERP) method, which is an electrophysiological technique that provides precise millisecond data about the timing of visual processes in response to an event or stimulus [21]. Studies have measured ERPs elicited in response to coloured stimuli that vary in their categorical relationship with each other, and category effects in the elicited ERP waveforms have been examined. The timing and polarity of ERP waveform components gives an indication of stage and type of processing related to that component. For example, the P1 ERP component is a component with a positive deflection that occurs roughly 100 ms after stimulus onset, and the P1 is thought to correspond to activity in early sensory stages of colour processing in the visual cortex, prior to visual awareness [15–17]. A number of ERP studies have claimed to find colour category effects throughout early ERP components such as the P1 and early-phase N1 whereby the categorical relationship of colour differences appears to modulate the amplitude or latency of these components [14,15,22,23]. Other studies have claimed that such colour category effects only occur in later ‘post-perceptual’ components such as the P2 [12] and P3 [13] which are thought to correspond to attention, stimulus evaluation, memory or decision making [24–26] and reach peak amplitude approximately 200 and 300 ms after stimulus-onset respectively.

Although there are now a number of ERP studies of colour category effects, the majority of these studies are plagued by an important stimulus issue: same- and different-category colour differences are equated in colour metrics which have known inhomogeneities [27–29]. For example, although the colour metrics used in category studies such as CIELUV, CIELAB and Munsell attempt to be perceptually uniform, inhomogeneities are known to exist within such spaces, and these manifest as areas of greater and lesser discrimination sensitivity. Early ERP components are known to be highly sensitive to the physical differences between stimuli [30,31]. Therefore, effects which have been labelled as ‘category effects’ could instead be due to the different-category colour differences being greater than same-category colour differences when equated in the colour metrics used in prior studies [12].

There have only been three ERP studies so far which cannot potentially be explained by stimulus issues and the findings of these studies disagree. Thierry et al. [15] compared ERP components elicited in response to colours in native Greek and native English speakers. Greek and English differs in the categorization of the colour blue; the Greek language contains an additional colour category dividing lighter and darker shades [32]. Observers were not required to attend to the colour of stimuli and instead focused on detecting whether coloured stimuli were a square or circle while ERPs were recorded. It was found that for Greek speakers, a blue colour difference which was different-category in the Greek language elicited a stronger visual mismatch negativity (vMMN) ERP component (around 160–230 ms), than colour differences which were the same category, with no such effect for English speakers. A similar ‘category’ effect was also found for Greek speakers in the P1 ERP component. Both the vMMN and P1 components are thought to be pre-attentive [33,34] arising in early-sensory stages of visual processing. The apparent category effect for Greek speakers cannot be explained by stimulus issues since the English speakers, for whom there was no such effect saw the same colours. However, one potential issue with the study has been identified by Clifford et al. [13] who argue that the ERP waveforms for Greek and English speakers suggest that the English speakers attended more to the colour differences than Greek observers because there appears to be an attention-related P3 component for the English but not Greek speakers. Therefore, stronger ‘pre-attentive’ ERP components for Greek than English speakers for certain colour differences could potentially be due to different amounts of attention to colour during the task rather than cross-linguistic differences in colour terms. Nevertheless, further evidence for the early effects of language was provided by Thierry and colleagues in a re-analysis of their original study [35] where they find that the strength of the category effect for the Greek speakers was modulated by the length of time the Greek speakers had lived in the United Kingdom (and therefore their familiarity with the English language). Specifically, category effects were weaker for Greek speakers who had lived in the United Kingdom for 18 months or longer compared with Greek speakers who had lived there for less than a year.

A second ERP study, conducted by Clifford et al. [13] investigated the time course of category effects for newly trained colour categories. Observers were trained to categorize a set of colours varying in hue and lightness into two new categories with new colour terms, and ERPs were then measured to colours which varied in their categorical relationship according to these newly trained terms. Within a block of trials, one hue was presented frequently (the standard) and two infrequently presented hues (the deviants) were either from the same trained category as the standard or from a different category. Observers were required to count the number of deviant stimuli and therefore attend to the colour differences. The categorical relationship of the deviant hues with the standard was found to modulate post-perceptual ERP components 350–600 ms after stimulus onset, rather than earlier stages of visual processing as in [15]. No such category effects were found for a separate sample of observers who were not trained to categorize the hues into new categories, or for either group in an untrained hue region. These effects cannot be explained by stimulus issues since category effects were only found for those who underwent category training yet all observers saw the same stimuli.

The third ERP study which cannot be explained by stimulus issues is that of He et al. [12]. They used the same task as Clifford et al., but tested for category effects related to the blue-green categorical distinction in English speakers. To address concern over stimulus issues, same- and different-category colour differences between the standard and deviant hues were equated in number of just noticeable differences (JNDs) rather than relying on other colour metrics. As in Clifford et al., category effects, indicated by significantly different ERP amplitude for deviants from a different-category to the standard than the same-category, were found only in post-perceptual components 230 ms after stimulus onset.

In sum, whilst two studies claim that colour terms only affect post-perceptual processing of colour when stimulus issues are controlled [12,13], another study which draws on cross-linguistic differences in colour terms claims that colour terms do affect early stages of colour processing [15]. Further research is needed to explore this apparent discrepancy. One possibility is that the documented early effects are due to cross-linguistic differences in attention and are not related to naming [14]. The studies also differ in their task: the study which claimed to find early category effects used a task where observers were required to attend to the shape not the colour of the stimuli, whereas the two studies which find later post-perceptual category effects required observers to attend the changes in colour directly. Therefore, another possibility is that early category effects (e.g., in P1 or vMMN) are only found when attention is directed away from colour when the processing of colour is more implicit.

In the current study, we use a task where observers were not required to attend to colour as in the above mentioned studies [14,15,22]. Participants focused on a fixation dot and responded when it changed shape [14]. However, rather than comparing speakers of different languages who differ in their colour terms, here we investigate the impact of differences in colour term usage for observers speaking the same language. Intra-language colour term use can vary substantially: A recent study in native American English speakers found only 31% of 330 colour samples were named the same by all participants despite constraining responses to just 11 basic colour terms [36]. Having intra- rather than inter- language comparisons means that any effect of language that we find is more likely to be due to colour term usage rather than other group differences such as task strategy that could arise from cognitive or cultural difference. Relevant to the present study, differences in colour naming will result in differences in colour categorization when the colour in question is in the boundary region between two colour categories. For example, in the boundary region a particular colour may reliably be named yellow by one observer and reliably as orange by another. When presenting this colour alongside a different colour named orange by both observers, the first observer will see two colours from different colour categories (yellow and orange), whereas the second observer will see two colours from the same category (orange and orange) even though the same colours are presented to both observers.

In the present study we used three colours: A green, a blue, and a boundary colour in between the two. We selected these colours because green and blue are often the colour terms applied to the largest number of colour samples by native English speakers [36,37], and it has been observed that the location of the boundary between these colour categories can vary across English speakers [12,38]. Neighbouring colours were separated by three JNDs. The JND data was collected in a prior study [12] in which observers’ sensitivity to colour difference was measured psychophysically using a 3-up-1-down staircase procedure. Participants who completed the JND measurements did not take part in the present study; past research has found that over-familiarity with colour stimuli stimuli due to prior threshold measurement can weaken category effects [9], which was undesirable for the present study. In the present study observers completed a passive ‘visual oddball’ task, whereby participants were presented with the boundary blue-green colour on the majority of trials (here called the ‘standard’ stimulus in line with prior oddball paradigm literature). Two groups of observers reliably named this boundary colour differently to each other–one group reliably named it blue and the other group as green. On a smaller number of ‘oddball’ trials, participants were presented with the blue and the green colours (the infrequent ‘deviants’) for which the two groups agreed in their naming. Accordingly, depending on how observers named the boundary colour, the blue and green deviant colours were either the same- or different-category to the boundary blue-green colour. We recorded and compared the ERPs elicited by each of these three stimuli to assess whether the categorical relationship of the standard and deviant colours modulated the amplitude of ERP components.

As in [15], attention to the colour of the stimuli was not required because our observers were tasked with making a manual response when a central fixation dot changed. The stimuli were presented simultaneously as pairs, with one colour presented above the fixation dot and therefore to the upper visual field (UVF), and another below the dot and to the lower visual field (LVF). We included this manipulation of visual field because prior work has shown that ERPs differ depending on which visual field a stimulus is presented to. The difference in ERPs generated in response to UVF and LVF stimuli is likely due to the retinotopic structure of visual cortex [34]. Specifically, visual change detection measured through ERPs has been shown to be more sensitive to changes in visual stimuli presented to the LVF compared to the UVF [14,34]. However, an unanswered question is whether this asymmetric category effect in the LVF compared to the UVF remains when stimuli differences are equated psychophysically, rather than in colour spaces, such as the Munsell system, e.g., [14]. In the present study we measured ERPs elicited to colour stimuli separated in JNDs for both LVF and UVF stimuli to further investigate the relationship between category effects and spatial location. After the ERP task, we measured whether the participants named the three colours as blue or green so as to locate the blue-green colour boundary individually for each observer. Identifying the category boundary in this manner was favoured over measuring colour naming across a larger range of stimuli because there are known effects on colour naming that arise from differences in the range of colour presented [39]. This approach also ensured that the names that the observers gave to the three colour stimuli were specifically relevant to their performance in the ERP oddball task. After the ERP task, participants named each colour 25 times so that we could establish the degree of colour naming consistency.

A passive visual oddball task often elicits a vMMN along with other early (e.g., P1) and late ERP components (e.g., N2). As outlined earlier, one prior study has found evidence for category effects in both the vMMN and P1 [15] and two other studies find category effects in later ‘post-perceptual’ ERP components (e.g., N2, [12]). In order to test the hypothesis that early category effects are found when attention is directed away from colours, we analysed the early ERP components elicited by our task for which category effects have previously been found. We also analysed the post-perceptual ERP components elicited by our task that have been implicated in category effects in studies where colour is focused on during the task, to test whether category effects in post-perceptual components remain when attention is directed away from the colours. Category effects were investigated by comparing ERP components elicited by same- and different-category deviants. In addition, the effect of naming was investigated by testing the effect of how consistently the standard was named on the size of the category effect: if colour naming affects colour processing then those who more consistently name the colours should show greater category effects. We also analysed the relationship between category effects and spatial location (UVF vs. LVF). Stronger category effects were expected because the visual system is more sensitive to stimuli in the LVF than those in the UVF [14,34].

## Methods

### Participants

Thirty-three native British English speakers (24 female; mean age = 21.3; *SD* = 2.96; range = 18–30), who were naive to the purpose of the study, took part. Participants were recruited from the University of Sussex. Data collection took place for six months starting June 2013. All participants were screened for colour vision deficiencies using the Ishihara test [40] and the City University Test [41]. Participants provided written informed consent and were compensated with cash or course credits. The study was approved by the Cluster-based Ethics Research Committee of Psychology and Life Sciences at the University of Sussex. An a priori power analysis of the effect size (*d* = 0.86) reported by Clifford et al. [14] showed that a sample size of *N* = 17 would achieve a power of > 0.95 to detect a significant category effect in early stages of cortical visual processing.

### Stimuli and set up

Participants were seated in a dark room, the only source of light was a 22" Diamond Plus 230SB CRT monitor (Mitsubishi, Tokyo, Japan; colour resolution: 8 bits∕channel; spatial resolution: 1024 × 768; refresh rate: 75 Hz), located 77 cm away from participants. Gamma correction was applied after measuring monitor primaries with a CRS ColorCal (Cambridge Research Systems, Rochester, UK). The CIE1931 chromaticity coordinates and luminance of the monitor primaries were (R: 0.626, 0.337, 14.24; G: 0.281, 0.614, 45.51; B: 0.151, 0.071, 5.28). All materials were prepared with e-Prime 2 (Psychology Software Tools, Inc.). Test stimuli were three isoluminant, isosaturated colours varying in hue in CIELUV space and presented on a grey background. The colours were taken from [12], who made psychophysical measurements of colour discrimination using a 3-up-1-down staircase procedure. Our adjacent colours were separated by three JNDs and the colours spanned the categories of blue and green (for colour chromaticity coordinates see Table 1). We used the same monitor set up as [12]. It was anticipated that the central boundary colour would be named blue by some participants and green by others. Note that the boundary colour is also referred to as the ‘standard’ due to a greater frequency of presentation of this stimulus in the oddball paradigm (see Design and Procedure below).

**Table 1:** Chromaticity coordinates (*x*,*y*,*Y* CIE1931) of test stimuli and background.

	*x*	*y*	*Y*
Boundary / Standard	0.222	0.333	9.21
Green deviant	0.237	0.380	9.21
Blue deviant	0.220	0.292	9.21
Background grey	0.313	0.329	20.37

### Design and procedure

#### Passive oddball task

**Fig 1:**
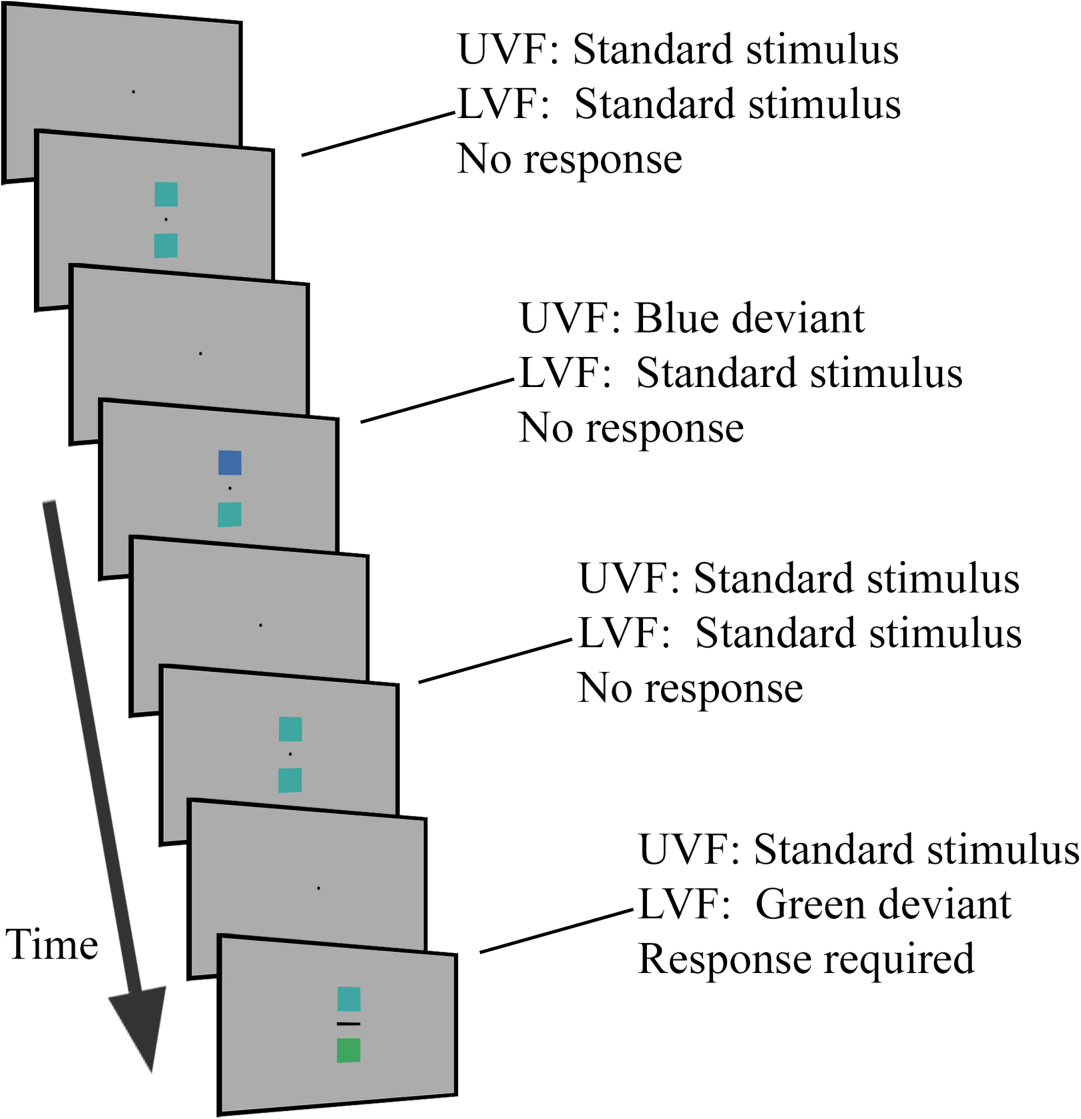
General task procedure of the passive visual oddball ERP task. On each trial a coloured square was presented to the UVF and another simultaneously to the LVF. Participants attended to a fixation dot and responded on those trials in which it changed shape. For the majority of trials both squares were the standard (boundary) stimulus. The remaining trials presented either a green or blue deviant stimulus to the UVF or LVF. Stimuli were presented for 200 ms with a randomised interstimulus interval of 1,000 ms ± 200 ms.

Participants first completed a passive visual oddball task. The stimuli and task procedure are illustrated in Fig 1. An oddball task presents the same stimulus on the majority of trials (referred to as the ‘standard’), while different ‘oddball’ stimuli are occasionally presented (referred to as ‘deviants’). On each trial there was the simultaneous presentation of two coloured squares (length: 1.93° visual angle) for 200 ms towards the centre of the screen and ordered vertically such that the space between them was equal to their size. This resulted in one square being presented towards the upper visual field (UVF) and the other the lower visual field (LVF; see Fig 1). For 90 trials in each block both upper and lower squares were the standard (boundary) colour. Half of the 20 deviant trials presented the blue deviant and the other half the green deviant, with equal probabilities shown in the upper or lower visual field. In each block the fixation dot (0.13° in diameter) remained in the centre of the screen and changed to a horizontal bar at the onset of 10 random trials (0.46° × 0.13°). Observers were asked to attend to the black fixation dot while the colour stimuli were presented above and below the dot. This design is referred to as passive because participants are not required to attend to or make decisions about the coloured stimuli. Participants were asked to respond quickly and accurately when the fixation changes took place by pressing the space key with both hands, and were told that the colours were not relevant to the task. A randomised interval ranging from 800 to 1,200 ms was used between trials. In each block the trial sequence was pseudo-randomised so that the first 8 trials were always standard trials and no consecutive deviant trials were allowed. In total there were 18 blocks of 110 trials.

#### Colour naming task

Following the ERP oddball task participants completed a colour naming task, whereby each of the three colour stimuli were presented individually 25 times in a randomised order. Participants were asked to indicate if the stimulus was green or blue by pressing the “c” or “m” key (counterbalanced across participants). Stimuli were presented as a coloured square (7.5° × 7.5°) in the centre of the screen and remained onscreen until a response had been made with an interstimulus interval of 1,500 ms. The same background grey was used as the oddball task.

### EEG recording and processing

EEG data was recorded and processed with NeuroScan SynAmps^2^ amplifiers and SCAN 4.3 software (NeuroScan/Compumedics, Inc.) at a digitizing rate of 500 Hz. A physical band-pass filter was applied to online recording (0.10–100 Hz). EEG was recorded from 62 electrode sites: FP1, FPz, FP2, AF3, AF4, F7, F5, F3, F1, Fz, F2, F4, F6, F8, FT7, FC5, FC3, FC1, FCz, FC2, FC4, FC6, FT8, T7, C5, C3, C1, Cz, C2, C4, C6, T8, TP7, CP5, CP3, CP1, CPz, CP2, CP4, CP6, TP8, P7, P5, P3, P1, Pz, P2, P4, P6, P8, PO7, PO5, PO3, POz, PO4, PO6, PO8, O1, Oz, O2, I1 and I2, using Ag-AgCl electrodes, as well as the average of the left and right mastoid references (re-referenced offline). Eye blinks and eye movements were monitored via one bi-polar horizontal electro-oculogram (EOG) channel located laterally of the canthi and one bi-polar vertical EOG channel located above and below the participant’s left eye. Impedance of each channel was reduced below 5kΩ prior to data collection. Following EEG recording, a zero phase-shift low-pass filter with amplitude cut off frequency of 30 Hz and 48dB/oct roll-off was applied to the data. The recorded EEG data were analysed as segments extending 800 ms after stimulus onset relative to a 100 ms pre-stimulus baseline, averaged over trials in each experimental condition. Trials were rejected as artefacts when voltage exceeded ±60 μV at any electrode. Criteria for artefact rejection were determined on the basis of previous research (e.g., [12]), from which ERPs were used to successfully investigate colour category effects. ERPs were generated by averaging EEG activities over trials time-locked to stimulus onsets.

## Results

### Colour naming task

The blue deviant was consistently named blue (*M* = 98.1%; *SD* = 4.5%) and the green deviant green (*M* = 98.6%; *SD* = 2.7%). As expected, naming of the standard was variable across participants: averaged across all trials it was named blue 48.7% (*SD* = 36.4%) of the time. However, the tendency for an individual to name the standard consistently green (green namers) or consistently blue (blue namers) was higher (*M* = 81.9%; *SD* = 16.7%).

### EEG passive oddball task

Two participants were excluded as they elicited strong alpha waves (8–13 Hz EEG rhythmic activity), which substantially contaminated the ERP waveforms and one participant was excluded due to an insufficient number of trials following EEG data processing. For the blue namers, a deviant trial consisting of the simultaneous presentation of the standard (i.e., named blue) as well as the blue deviant is a same-category deviant trial. For the green namers this is a different-category trial. This pattern is reversed on trials that present the green deviant. Classifying stimuli in this manner as same- or different-category on the basis of an individual’s naming was previously adopted to analyse colour category effects in fMRI [38] and ERP data [12]. Data were combined across all participants (*N* = 30) with three conditions: 1. ERPs elicited to the standard (i.e., both squares are the boundary colour); 2. ERPs elicited on same-category deviant trials; 3. ERPs elicited on different-category deviant trials.

**Fig 2:**
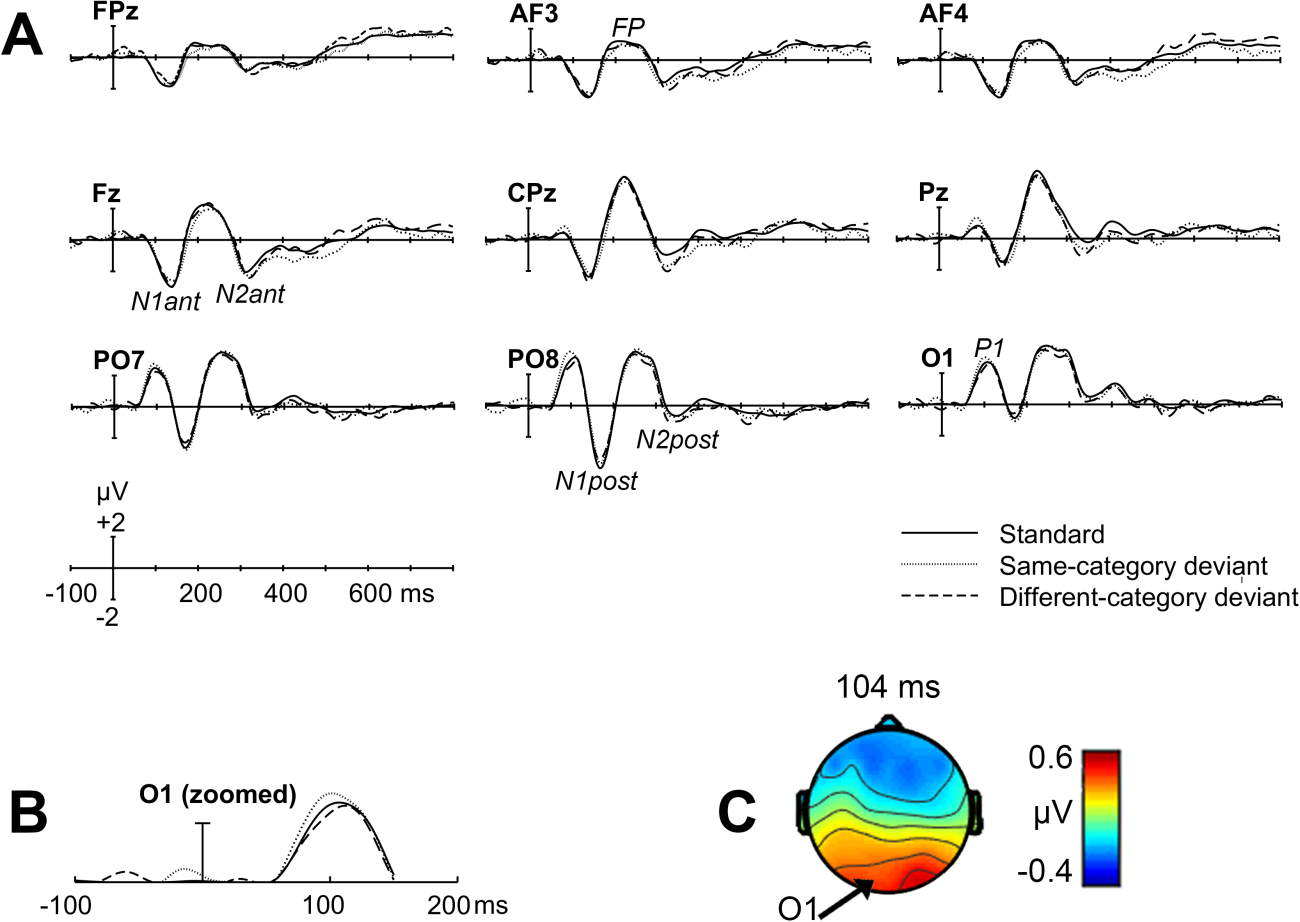
Grand-averaged ERP waveforms elicited in response to standard and deviant colours presented to the lower visual field. (A) Waveforms elicited for 800 ms following stimulus onset summarised over nine representative electrode locations. Stimuli were classified as same- or different-category to the standard for each individual based on their naming of the standard stimulus as blue or green. Electrode locations are provided towards the top of the y-axes. ERP components (e.g., P1) are labelled on one waveform each. N1ant denotes the anterior N1 component, N1post denotes the posterior N1, FP denotes frontal positivity, N2ant denotes the anterior N2, and N2post denotes the posterior N2. (B) A category effect in P1 over a refined time period (94–104 ms): The different-category deviant elicited a significantly more negative mean amplitude than the same-category deviant displayed here at a representative electrode (O1). (C) Topographic map showing the amplitude difference (same- minus different-category deviant of the the P1 effect. The arrow shows the location of representative electrode O1).

The data were analysed in two ways. Firstly, the data were analysed with mixed ANOVAs containing the factors of category with three within-subjects levels (standard, same-category, and different-category), and the factor of group with two between-subjects levels (blue namers vs. green namers), see Table 2. A category effect is demonstrated by a significant main effect of category, with subsequent post-hoc analysis revealing a significant difference between the ERP responses elicited to the different- and same-category deviants. This finding would suggest that a particular ERP component is sensitive to the categorical relationship between the stimuli. If the category effect is reliable then it should be found for both blue namers and green namers and there should be no significant interaction between category and group. Secondly, the data were analysed with linear regressions to investigate the relationship between naming consistency of the standard and the degree of amplitude difference elicited by the same- and different-category deviants. In other words, does the categorical relationship between the stimuli have a greater effect on ERPs for participants who more consistently name the standard compared to participants who less consistently name the standard? Trials requiring a manual response were excluded from all analyses to avoid contamination of ERPs from electrical activity arising from the execution of a motor response. Electrode locations were chosen for each ERP component separately to reflect sites where activity was maximal. Greenhouse-Geisser corrections were applied to those instances in which the assumption of sphericity had been violated and significant main effects in the ANOVAs were followed up with pairwise comparisons comprising Fisher’s least significant different (LSD) post-hoc test. The analysis focuses on mean amplitude (*μ*V). Peak latency was not analysed because it was not possible to discern reliable peaks across a suitable number of participants. ERP waveforms for LVF stimuli are presented in Fig 2A. The UVF waveforms are presented in S1 Fig.

**Table 2:** Summary of analyses of ERP components from two-way mixed ANOVAs containing the factors of category (within-subjects; 3 levels: standard, same- and different-category deviant) and group (between-subjects; 2 levels: blue namers vs. green namers), N = 30.

ERP component	Time window (ms)	Standard mean amplitude (SEM)	Same-category deviant mean amplitude (SEM)	Different-category deviant mean amplitude (SEM)	Category *F* value (η_p_^2^)	Group *F* value (η_p_^2^)	C × G *F* value (η_p_^2^)
P1	94–104	2.63 (0.30)	2.70 (0.36)	2.84 (0.36)	0.28 (.010)	0.02 (.001)	3.13 (.100)
			3.06 (0.31)	2.31 (0.40)	3.88 (.122)*	0.06 (.002)	0.12 (.004)
Anterior N1	130–140	-2.91 (0.27)	-2.62 (0.38)	-2.80 (0.44)	0.43 (.015)	0.05 (.002)	2.22 (.073)
			-2.46 (0.45)	-2.92 (0.40)	0.97 (.034)	0.48 (.017)	0.01 (.000)
Posterior N1	164–174	-3.08 (0.48)	-2.82 (0.54)	-2.78 (0.56)	0.65 (.023)	0.33 (.012)	1.56 (.053)
			-3.02 (0.50)	-2.62 (0.55)	1.47 (.050)	0.18 (.006)	0.52 (.018)
Frontal Positivity	220–260	1.14 (0.35)	1.19 (0.41)	1.47 (0.42)	0.61 (.021)	4.28 (.133)*	0.71 (.025)
			0.98 (0.57)	1.05 (0.38)	0.07 (.002)	3.77 (.119)	0.34 (.012)
Anterior N2	316–356	-1.41 (0.25)	-1.85 (0.38)	-1.95 (0.38)	1.10 (.038)	0.22 (.008)	0.96 (.033)
			-1.99 (0.43)	-2.02 (0.32)	1.81 (.061)	1.58 (.053)	0.01 (.000)
Posterior N2	330–360	-0.19 (0.39)	-0.68 (0.45)	-0.47 (0.47)	0.94 (.033)	0.38 (.014)	1.06 (.036)
			-0.35 (0.38)	-0.64 (0.45)	1.43 (.049)	0.99 (.034)	0.93 (.032)

Notes: For each ERP component the values in the upper row correspond to deviants presented to the upper visual field and the values in the lower row to deviants presented to the lower visual field. C x G: Interaction between factors of category and group. P1 electrode locations: PO8/PO7/PO6/PO5/PO4/PO3/O1/O2. Anterior N1 electrode locations: F1/Fz/F2/FC1/FCz/FC2/C1/Cz/C2. Posterior N1 electrode locations: PO8/PO7/PO6/PO5. Frontal positivity electrode locations: AF4/AF3. Anterior N2 electrode locations: F1/Fz/F2/FC1/FCz/FC2/C1/Cz/C2/CP1/CPz/CP2. Posterior N2 electrode locations: PO8/PO7/PO6/PO5/PO4/PO3.

* p < .05. Mean amplitude values denote μV.

In line with prior research, there were no category effects when deviants were presented to the upper visual field [14,34]. For deviants presented to the lower visual field we found a significant main effect of category in P1 over a 10 ms time window (94–104 ms), *F*(2,56) = 3.88, *p* = .026. Post hoc analysis revealed that the category effect occurred because the P1 elicited by the different-category deviant (*M* = 2.31 μV; *SD* = 1.69 μV) was significantly more negative than that elicited by the same-category deviant (*M* = 3.04 μV; *SD* = 1.69 μV; *p* = .025). The standard (*M* = 2.61 μV; *SD* = 1.63 μV) fell numerically, but not significantly, between the two. Note that this effect in P1 was highly refined with regards to its timing; over a longer time window (80–120 ms) mean amplitudes did not differ significantly. There were no other category effects in subsequent ERP components for stimuli presented to the lower visual field. There was a significant effect of group in the frontal positivity when deviants were presented to the upper visual field, *F*(1,28) = 4.28, *p* = .048. This was due to blue namers (*M* = 1.92 μV; *SD* = 1.74 μV) exhibiting greater frontal positivity than green namers (*M* = 0.48 μV; *SD* = 2.13 μV), rather than a category effect. The regression analyses found no significant relationship, for deviants presented to the UVF, between naming consistency and the mean amplitude difference between the different- and same-category deviants (i.e., a category effect) in any ERP components (see Table 3). However, for deviants presented to the LVF, a significant relationship between naming consistency and category effect was found in the frontal positivity (220–260 ms). Here, naming consistency significantly predicted the difference in mean amplitude between the different- and same-category deviants, and the trend was for the different-category deviant to elicit more negativity than the same-category deviant for more consistent namers.

**Table 3:** Linear regression analyses applied to multiple ERP components modelling the relationship between naming consistency of a boundary blue-green colour (i.e., the standard stimulus) and the difference in mean amplitude (μV) elicited by a same- and different-category deviant colours presented to the UVF or LVF (*N* = 30).

	Upper visual field				Lower visual field			
	B	SE B	β	R^2^	B	SE B	β	R^2^
P1								
Constant	3.07	1.75			-1.93	1.44		
Naming consistency	-0.04	0.02	-0.31	0.10	0.01	0.02	0.16	0.02
Anterior N1								
Constant	-0.16	1.77			-0.10	2.22		
Naming consistency	0.00	0.02	0.00	0.00	0.00	0.03	-0.03	0.00
Posterior N1								
Constant	3.05	1.71			2.55	1.60		
Naming consistency	-0.04	0.02	-0.32	0.10	-0.03	0.02	-0.25	0.06
Frontal positivity								
Constant	1.49	1.79			5.90	2.17		
Naming consistency	-0.02	0.02	-0.13	0.02	-0.07	0.03	-0.46*	0.21
Anterior N2								
Constant	-0.23	1.66			1.45	2.03		
Naming consistency	0.00	0.02	0.02	0.00	-0.02	0.02	-0.14	0.02
Posterior N2								
Constant	0.74	1.72			-0.54	1.53		
Naming consistency	-0.01	0.02	-0.06	0.00	0.00	0.02	0.03	0.00

Note: All values rounded to 2 decimal places.

* *p* < .05

### Behavioural performance analyses

Hit rates from participants included in the ERP analysis (*N* = 30) for target trials were very high (*M* = 99.8%; *SD* = 0.30%) suggesting participants attended to the fixation dot throughout testing. False alarm rates were very low (*M* = 0.04%; *SD* = 0.03%). Mean response time to targets was 389 ms (*SD* = 28.8 ms).

## Discussion

We measured ERPs on a passive oddball task to blue and green colours equated in JNDs that varied in their categorical relationship for two groups of observers who differed in colour naming. We found evidence for a category effect 100 ms after stimulus onset in the P1 component, whereby a different-category colour elicited a more negative electrophysiological response compared with a colour from the same category. Note that this is not simply an effect of hue on P1 because the stimuli were deliberately grouped by category rather than by hue (the same- and different-category hues were different for the two groups of observers who differed in colour naming). The categorical relationship between colours and the variation in the way that observers consistently named the boundary colour was also found to affect neural activity, specifically over frontal sites around 250 ms. At this stage of visual processing, naming consistency predicted the difference in amplitude elicited by the same- and different-category deviants and the trend was for the mean amplitude elicited to a different-category colour to be more negative when naming consistency (and therefore the categorical distinction between the stimuli) was higher. These findings suggest that colour language affects colour processing at both an early stage as well as a later post-perceptual stage of visual processing.

The P1 has been reported to originate from sources in dorsal extrastriate cortex of the middle occipital gyrus and the ventral extrastriate cortex of the fusiform gyrus [42,43]. While the P1 is known to be sensitive to the physical characteristics of stimuli, such as size [30], luminance [31], and spatial location [44], as well as attention, for a review see [45], there continues to be debate about whether this early stage of visual processing can be penetrated by cognitive systems, such as language. The data we report supports the claim that language can affect neural activity in this early stage of visual processing [14,15,46]. The P1 component elicited by the colour stimuli in the present study likely corresponds to unconscious, pre-attentive processes because participants directed their attention towards a fixation target rather than the colour stimuli [45]. We found that mean amplitude of P1 was more negative when it was elicited to a deviant colour from a different category compared to a deviant colour from the same category as the standard. The finding of greater negativity elicited to a different- compared to a same-category deviant has been reported previously on visual oddball tasks in the form of visual mismatch negativity (vMMN; [47]). The vMMN has been suggested to arise from the automatic processing of unattended visual stimuli and as a marker of low-level, pre-attentive perceptual processing [14,33]. It is thought to be characterised by a posterior distribution and occur from around 100–250 ms [14,48,49]. A question here is whether the finding of greater negativity elicited to the different-category deviant in the present study in P1 over posterior sites should be viewed as a category-related vMMN response [14,48]. A principal difference we report is that this effect was limited to a refined stage around peak amplitude of P1, rather than extending over a longer time period [14,15]. The effect we report may be relatively small and within a more refined period than previous studies due to the current study using stimuli equated in JNDs, which may have resulted in subtler differences in ERP amplitudes.

Further evidence that the categorical relationship between colours plays a role in the way they are processed was found over frontal sites from 220–260 ms (we refer this as frontal positivity). Here, we found a relationship between whether a boundary blue-green colour was more or less consistently named with the same colour term and the difference in mean amplitude elicited by a colour from the same category compared to a different category. A similar post-perceptual category effect was found in He et al.’s study [12] which also used blue and green stimuli equated in JNDs. In their study [12] there was a significant category effect over frontal sites from 210–260 ms, which was characterised by the different-category colour eliciting a greater amplitude than a same category-colour. The effect we report is different in that we found that a significant proportion of the variance associated with the different mean amplitudes elicited to our colour stimuli is explained by the degree that observers reliably named (and therefore categorised) the stimuli. For observers who more reliably categorised the stimuli the different-category deviant tended to elicit a more negative ERP deflection than the same-category deviant.

For both P1 and the frontal positivity, the effects we report were specifically found for stimuli presented to the LVF, rather than the UVF. This provides further support for those findings from ERP studies that have likewise compared electrophysiological activity elicited to stimuli presented to the lower and upper visual fields. For example, it has been shown that the vMMN is larger for colour patches presented to the LVF [14], and may even be absent for colour patterns presented to the UVF [34]. Clifford et al. [14] previously found colour category effects only in the LVF. Here we replicate this finding and extend it to with stimuli specifically equated in JNDs.

In the present study we found tentative evidence for a colour category effect in P1. This suggests that the categorical relationship between colour stimuli is registered in early sensory stages of visual processing. This effect was governed by the way that participants named the colours. The effect in P1 is consistent with some behavioural evidence suggesting that the way people name and categorize colours affects performance on colour tasks. For example, it has been shown that native Russian speakers, who like Greek speakers divide blues into two basic lighter and darker categories, exhibit faster reaction times on a colour matching task when distractor stimuli come from a different blue category compared to the target [7]. For English speakers, who do not categorise blues in this manner, no such effect was found. The authors of this study suggest this is indicative of an interaction between lower-level perceptual processing and language. However, from this behavioural work the timescale of this interaction and whether this is truly low-level is not clear. If the category effect we report in P1 is the cortical basis that underpins observable differences in performance on colour tasks, then our data support claims that language interacts with low-level stages of visual processing, e.g., [7,14,15,46]. However, in the present study we also observed a relationship between colour naming consistency and colour processing around 250 ms in the frontal positivity. This may instead implicate an attentional, top-down, post-perceptual component to category effects. In other words, it is plausible that this activity could be responsible for previously reported behavioural category effects, such as [7] and [9], without needing to invoke an early category effect at all. Electrophysiological support for this ‘post-perceptual’ account of category effects was reported by [12], who find no early, low-level category effects in ERPs for colours equated in JNDs on a visual oddball task. The effect we report in P1 is evidently at odds with this finding and isolating the cause of these different outcomes is clearly important for understanding the relationship between language and visual processes.

One solution to these contradictory findings may reside in differences in how much colour is attended during the task. In the current study participants were tasked with attending to an infrequently-changing fixation dot so the colour stimuli were not directly attended. In [12], participants directly attended to the colour of the stimuli. It may be the case that colour terms lead to category effects in early sensory processes when colour is not explicitly attended but not when colour is attended. This hypothesis may not seem logical, why would the categorical relationship between colours be encoded when colour is not explicitly attended and colours are processed to a greater degree outside of awareness? One possibility is that categorical processing is more greatly recruited under conditions of greater stimulus uncertainty. There is some support for this view; colour category effects were found to be stronger in participants on a behavioural task when they were less familiar with the colour stimuli compared to participants who were highly trained with the stimuli [9]. It may be that the visual system evolved this way so that a change in the visual scene is processed more categorically (e.g., threat versus no-threat) when outside of direct focus in order to increase the chance that danger is more readily perceived (c.f. [19]). A limitation of our design is that we were not able to test this possibility directly. Future research should be able to provide clarity here by using a blocked within-subjects design and comparing ERP amplitudes elicited by deviant stimuli in an oddball task in a passive condition (as in our design) to the amplitudes elicited to the same deviants in an active condition (whereby participants directly attend to colour change as in [12]). If it is the case that there is greater categorical processing to changes in the visual environment occurring outside of direct attention one would expect to find category effects in early stages of visual processing in the passive condition but not in the active condition.

Another area to consider is the direction of the relationship we report between colour naming and the category effects. Thus far we have considered language and the way people name colours as a mechanism that may penetrate colour processing, that is to say language affects perception. However, it may be the case that physiological differences in the visual system across individuals give rise to differences in colour naming. In other words, category effects could be the cause rather than result of the group differences in colour naming. Cone pigment [50], macular pigmentation [51], the optical density of retinal photopigments [52], eye pigmentation [53], as well as the relative number of L and M cones [54], are known to vary across individuals and might account for such differences. However, others have not found a link between physiological differences and colour naming. For example, it has been shown that individual differences in unique hue settings (pure examples of the terms red, green, blue and yellow) do not relate to individual differences in the sensitivity of the spectral sensitivities of the cones [55,56]. Further, cross-cultural differences in colour naming cannot readily be explained by physiological differences in the visual system [57,58]. A task for future research will be to clarify the relationship between these low-level physiological attributes and colour naming.

It has previously been shown using fMRI that explicit naming of attended colours modulates activity at V4 and VO1 [59], although representation in these regions was found to be non-categorical when attention was directed away from the colours. Likewise, several fMRI studies have failed to find an effect of colour categories on activity in visual cortex when colours are passively viewed [38,60]. However, our result does suggest a relationship between colour naming and early sensory processes even when colour changes do not need to be attended. Further investigation of the neural basis of our effect at P1 and the neural representation of colour categories will be important to establish the conditions under which language really does interact with our early sensory visual processing and the underlying mechanisms of such an effect. Shedding light on this question has the potential to address more fundamental issues about how colour is perceived, the source of individual differences in colour perception, as well as the degree to which language has the capacity to affect the way we see the world.

## Supporting information

S1 Fig Grand-averaged ERP waveforms elicited in response to standard and deviant colours presented to the upper visual field. (DOCX)
