# Supplementary material for: Colour categories are reflected in sensory stages of colour perception when stimulus issues are resolved
Source: PLoS One. 2017 May 25;12(5):e0178097. doi: 10.1371/journal.pone.0178097 (PMC5444794; doi:10.1371/journal.pone.0178097)
Supplement: S1 Fig — (DOCX) [file pone.0178097.s001.docx]

**Supporting Information**

Colour categories are reflected in sensory stages of colour perception when stimulus issues are resolved

Lewis Forder,^1,3^ Xun He,^2^ Anna Franklin^1,4^

^1^The Sussex Colour Group, School of Psychology, University of Sussex, BN1 9RH, UK

^2^Cognition and Cognitive Neuroscience Research Centre, Department of Psychology, Bournemouth University, Poole, BH12 5BB, UK

^3^email: [L.Forder@sussex.ac.uk](mailto:L.Forder@sussex.ac.uk)

^4^email: [anna.franklin@sussex.ac.uk](mailto:anna.franklin@sussex.ac.uk)

| 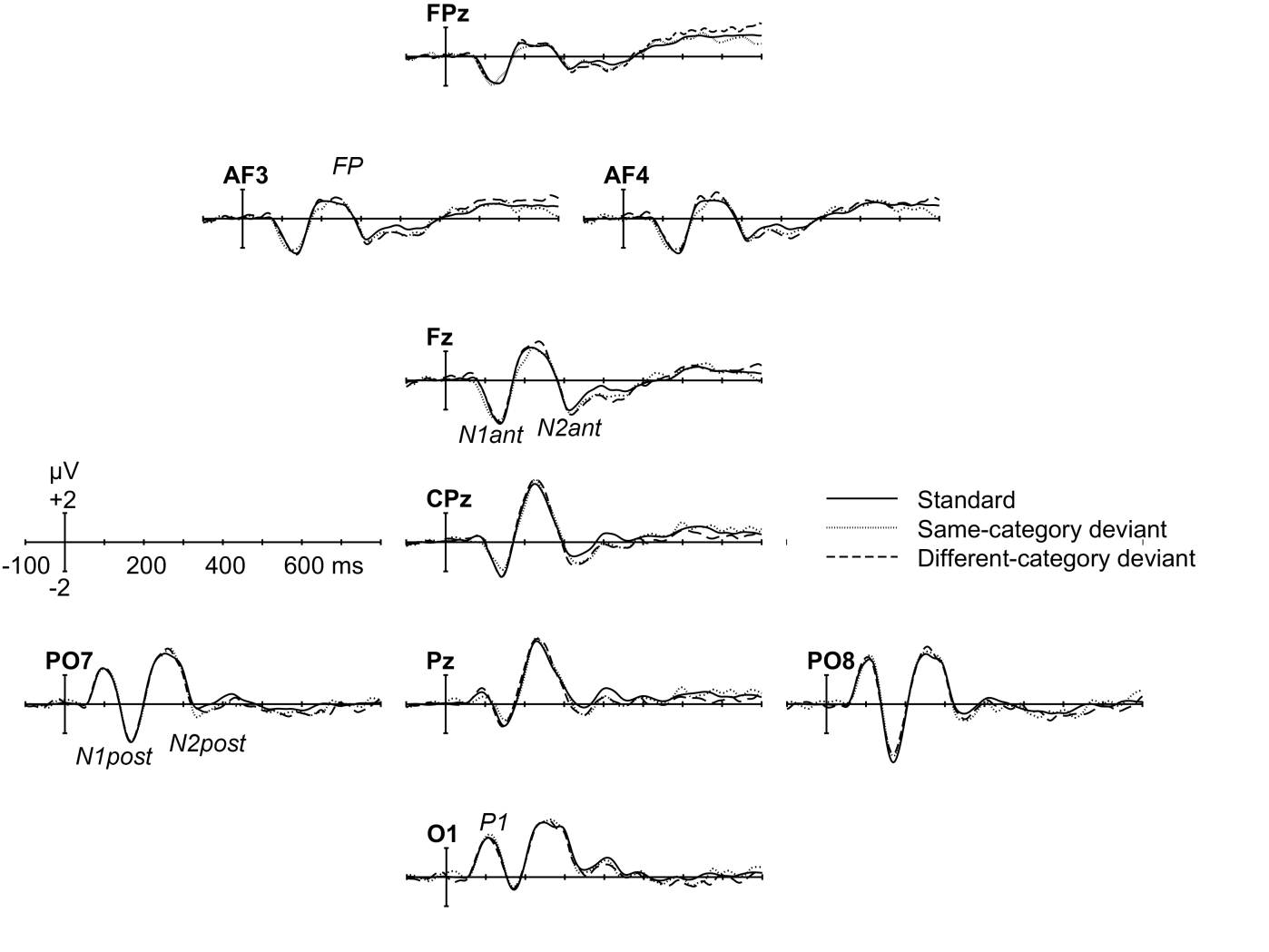 |
| --- |
| **S1 Figure. Grand-averaged ERP waveforms elicited in response to standard and deviant colours presented to the upper visual field.** (A) Waveforms elicited for 800 ms following stimulus onset summarized over nine representative electrode locations. Stimuli were classified as same- or different-category to the standard for each individual based on their naming of the standard stimulus as blue or green. Electrode locations are provided towards the top of the y-axes. ERP components (e.g., P1) are labelled on one waveform each. N1ant denotes the anterior N1 component, N1post denotes the posterior N1, FP denotes frontal positivity, N2ant denotes the anterior N2, and N2post denotes the posterior N2. |
